# RTS,S/AS01 malaria vaccine pilot implementation in western Kenya: a qualitative longitudinal study to understand immunisation barriers and optimise uptake

**DOI:** 10.1186/s12889-023-17194-2

**Published:** 2023-11-18

**Authors:** Jenna Hoyt, George Okello, Teresa Bange, Simon Kariuki, Mohamed F. Jalloh, Jayne Webster, Jenny Hill

**Affiliations:** 1 Department of Clinical Sciences, Liverpool School of Tropical Medicine, Liverpool, UK; 2 Kenya Medical Research Institute/Centre for Global Health Research, Kisumu, Kenya; 3 Global Immunization Division, Centers for Disease Control and Prevention, Atlanta, GA USA; 4 Disease Control Department, London School of Tropical Medicine and Hygiene, London, UK

**Keywords:** Immunisation, Malaria vaccine, RTS,S/AS01, Caregiver, Uptake, Longitudinal studies, Kenya

## Abstract

**Background:**

Malaria is a significant public health threat in sub-Saharan Africa, particularly among children. The RTS,S/AS01 malaria vaccine reduces the risk and severity of malaria in children. RTS,S/AS01 was piloted in three African countries, Ghana, Kenya and Malawi, to assess safety, feasibility and cost-effectiveness in real-world settings. A qualitative longitudinal study was conducted as part of the feasibility assessment. This analysis explores RTS,S/AS01 vaccination barriers and identifies potential motivators among caregivers in three sub-counties in western Kenya.

**Methods:**

A cohort of 63 caregivers with a malaria vaccine eligible child was interviewed at three time points over 24 months. A sub-set of 11 caregivers whose eligible children were either partially or non-vaccinated were selected for this sub-analysis. The 5A Taxonomy for root causes of under-vaccination was used to organise the inductively-coded data into categories (*awareness, acceptance, access, affordability,* and *activation*) and identify the factors influencing uptake across caregivers. A trajectory analysis was conducted to understand changes in factors over time within each caregiver experience. Caregiver narratives are used to illustrate how the factors influencing uptake were interrelated and changed over time.

**Results:**

Lack of awareness, previous negative experiences with routine childhood immunisations and the burden of getting to the health facility contributed to caregivers initially delaying uptake of the vaccine. Over time concerns about vaccine side effects diminished and anticipated vaccination benefits strongly motivated caregivers to vaccinate their children. Persistent health system barriers (e.g., healthcare provider strikes, vaccine stockouts, negative provider attitudes) meant some children missed the first-dose eligibility window by aging-out.

**Conclusions:**

Caregivers in this study believed the RTS,S/AS01 to be effective and were motivated to have their children vaccinated. Despite these positive perceptions of the malaria vaccine, uptake was substantially hindered by persistent health system constraints. Negative provider attitudes emerged as a powerful deterrent to attending immunisation services and hampered uptake of the vaccine. Strategies that focus on improving interpersonal communication skills among healthcare providers are needed.

**Supplementary Information:**

The online version contains supplementary material available at 10.1186/s12889-023-17194-2.

## Introduction

Despite a promising decline in malaria deaths in children under 5 years of age over the past two decades, malaria remains a significant burden in sub-Saharan Africa (SSA) [1]. In Kenya, even with effective preventive measures available, cases of severe malaria are concentrated in children under 5 years of age, with *Plasmodium falciparum* responsible for most infections and deaths [1, 2]. After decades of research to develop a malaria vaccine, the RTS,S/AS01 malaria vaccine emerged as a promising candidate offering a 40% reduction in malaria episodes and a significant reduction in life-threatening severe malaria caused by *P. falciparum* [3, 4]. The vaccine requires four doses initiated from 5 months of age and delivered through the existing childhood immunisation programme. In 2018, the World Health Organization (WHO) launched a pilot implementation in Ghana, Kenya and Malawi to further evaluate the safety, effectiveness, and real-world implementation of a malaria vaccination programme, including to understand the feasibility and uptake of the four-dose primary series vaccine schedule [5].

Vaccination barriers have been studied extensively, but to a lesser extent in the context of SSA. A systematic review revealed that barriers to uptake of routine childhood vaccinations in SSA occur at the caregiver, community, and health systems level. Low caregiver knowledge, lack of access to services, poor healthcare provider attitudes and distrust in immunisation programmes were identified as prominent barriers [6]. Existing evidence suggests that whilst community perceptions towards a potential malaria vaccine are positive [7–9], lack of information about the vaccine and concerns about side effects could hamper receptivity. In addition, health system barriers and low-quality immunisation services, such as lack of supplies and poor healthcare provider attitudes, have been identified as potential obstacles to uptake of the malaria vaccine [7, 10, 11].

Recently there has been interest in how vaccine hesitancy - defined as *‘delay in acceptance or refusal of vaccination despite availability of services’* [11] - may influence vaccination delays and refusals, amid fears that hesitancy may pose a threat to vaccination programmes across SSA [12]. A scoping review exploring the role of vaccine hesitancy in Malawi, Kenya and Ethiopia concluded that vaccine hesitancy was driven by a complex interplay of factors at the individual, community and health systems level, but noted that there is limited data from SSA contexts [13]. Furthermore, it has been highlighted that existing models used to conceptualise vaccine hesitancy may not adequately account for contexts in which health system constraints are prominent, which could lead to misclassification of the root causes of under-vaccination in some populations [14]. Developing appropriate strategies to optimise uptake of the malaria vaccine will require a nuanced understanding of the different factors influencing under-vaccination, including the identification of hesitant attitudes towards the vaccine itself.

Pilot implementation of the malaria vaccine was overseen by the Malaria Vaccine Implementation Program (MVIP), comprising several diverse stakeholders at the global and country levels. As part of the malaria vaccine programme evaluation (MVPE), a qualitative longitudinal study (QLS) was initiated shortly after the launch of the malaria vaccine pilot implementation in western Kenya with the broad aim of understanding changes in factors that may influence supply and demand for the vaccine over a 2-year period. The full findings and design of the overall programme evaluation will be published elsewhere. However, a specific focus of the QLS was to understand how community members received information about the malaria vaccine and the factors that influenced demand for and uptake of the vaccine over time. This paper presents the experiences of caregivers of partially or non-vaccinated children and explores the factors that hindered or motivated uptake over time.

## Methods

### Study design

A cohort of 63 primary caregivers of children eligible for the RTS,S/AS01 malaria vaccine was recruited for the QLS to explore the social and contextual factors that may affect the introduction and uptake of the vaccine in western Kenya during the pilot introduction of the vaccine in the routine immunisation programme. The caregiver cohort was interviewed at three time points over a 2-year period to understand perceptions, experiences, and uptake of the four-dose vaccine schedule for children. In addition, the QLS considered whether vaccine hesitancy influenced uptake and completion of the four doses.

This sub-analysis focuses on a subset of 11 caregivers whose children were under-vaccinated, that is, either partially or non-vaccinated, with the malaria vaccine and explores their experiences and decisions over time in relation to uptake of the malaria vaccine. The QLS approach acknowledges that the personal, social and health system contexts within which caregivers make decisions about vaccines are dynamic, and as such, a longitudinal design enables a more nuanced interpretation of decisions made throughout the caregiver journey [15, 16]. Reporting of this study follows the guidelines outlined by the standards for reporting qualitative research (SRQR) Additional file 1 [17].

### Study sites

The QLS was conducted in three sub-counties in western Kenya, Muhoroni, Funyula and Homa Bay situated in Kisumu, Busia and Homa Bay counties, respectively. The three sub-counties were purposively selected from 23 randomly selected implementing clusters in the MVIP pilot programme to represent variations in malaria prevalence (“low” < 10, “medium” 10 - < 20, “high” =  > 40), geography, and socio-cultural factors (Table 1). Three wards were purposively selected from the 4–5 electoral wards within each sub-county to represent low (< 65%), medium (65–75%), and high (> 75%) measles coverage, which was used as a proxy indicator for health system capacity and access to immunisation services [18]. For example, among the three communities selected in Muhoroni, the lowest measles coverage was 63.9% and the highest coverage was 76.5%. One sub-location was then randomly selected from within each ward, providing a total of nine study communities. A sub-location is the smallest administrative unit in Kenya each served by at least one public health facility which is connected to a network of community health units staffed by community health volunteers (CHVs).

**Table 1:** Characteristics of the QLS study sub-counties

	**Muhoroni**	**Funyula**	**Homa Bay**
*Community*	*C10, 11, 12*	*C13, 14, 15*	*C16, 17, 18*
Malaria prevalence^a^	9.9	41.3	16.6
Rural/urban	Rural	Rural	Urban/peri-urban
Ethnicity	Luo	Luhya	Luo
Measles coverage (range, %)	63.9–76.5	59.2–72.3	64.7–103.2
Migration & accessibility context	Difficult terrain resulting in seasonal accessibility issues	Cross border mobility due to proximity with Uganda	Movement out of urban townships to rural areas during COVID-19

^a^*Plasmodium Falciparum* prevalence among children < 5 years of age

### RTS,S/AS01 vaccination schedule

The malaria vaccine was delivered through routine immunisation programmes alongside other childhood vaccines in all public and private health facilities in the vaccinating clusters. The vaccine schedule adopted in Kenya was three primary doses given to children at 6, 7, and 9 months of age and a fourth dose at 24 months. During the national training workshop, the Kenyan Ministry of Health broadened the eligibility for the first dose to between 6 and less than 12 months of age, with the second and third doses at least 1 month apart, and the fourth dose from 24 months, with an upper age limit of 3 years.

### Contextual factors

During the 2-year study, several contextual factors impeded the delivery and uptake of the malaria vaccine in the study sites. There were recurrent healthcare provider strikes in all three sub-counties at multiple time points. Population mobility affected communities for different reasons which meant that some caregivers changed their health care seeking from vaccinating clusters to non-vaccinating clusters (Table 1). Cross border movement from Funyula sub-county into Uganda was widely reported as was movement out of urban townships in Homa Bay to rural areas during the first wave of the COVID-19 pandemic, which arrived in Kenya in March 2020. Although health facilities in vaccinating clusters continued to provide services throughout the pandemic, changes were made to how immunisation services were delivered, such as restricting the number of clients and cessation of group health education sessions. Accessibility issues during the rainy season were common in Muhoroni. Prior to the launch of the vaccine in Kenya, there was limited community sensitisation and communication, due to funding constraints. Information available in the community about the vaccine emanated mainly from sensitisation activities conducted by MVIP evaluation teams, media coverage of the vaccine during the introduction phase, interactions with healthcare providers, and personal encounters with peers (George Okello, personal communication).

### Caregiver selection and recruitment

In each of the nine study communities, seven caregivers of RTS,S/AS01 eligible children aged 6–12 months were recruited (*n* = 63) to ensure a minimum number of five per community taking into account lost to follow up in subsequent rounds. The first two caregivers were purposively selected if their child had received dose-1 of the malaria vaccine, to ensure at least two caregivers who had accessed the vaccine were included in each community. The remaining five were randomly selected from lists of caregivers of dose-1 eligible children compiled by CHVs residing in each community. By the second interview, 10 caregivers had been lost-to-follow-up and were replaced with purposively selected caregivers from the same community who had a child eligible for dose-2 i.e., aged at least 7 months. By the third interview, 55 caregivers remained in the cohort, eight had been lost-to-follow-up. Caregivers with a minimum age of 15 years were eligible. Study staff approached prospective participants at their homes where they provided the study information, including the longitudinal nature of the study, and written informed consent was sought.

### Data collection

In-depth interviews (IDIs) with caregivers in the cohort were conducted at three time points over a 24-month period to capture completion of the four-dose schedule: Interview 1 (September 2019–January 2020) to capture receipt of the dose-1, interview 2 (September – December 2020) to capture receipt of doses 2 and 3, and interview 3 (March to August 2021) to capture receipt of the dose-4. An iterative approach was used for the topic guide such that it was modified following each round of interviews to ensure that new and emerging themes or contexts were captured in subsequent interviews. The guide explored the following areas: 1) health context – including childhood health concerns, past immunisation experiences, household decision-making; 2) perceptions of malaria – risk, prevention behaviours, care seeking; 3) perceptions of the malaria vaccine – including exposure to messages, knowledge, experience of the vaccine, side effects, information received from providers; 4) caregiver experiences with uptake of, and adherence to, the malaria vaccine – including opportunities, constraints, motivators; 5) treatment seeking for malaria illness and use of long lasting insecticide-treated nets. The topic guides for the second and third interviews included perceptions of COVID-19, any changes in health seeking behaviour related to the pandemic such as curfews and cessation of movement and/or social gatherings, and experiences of accessing immunisation services during the pandemic.

IDIs were conducted either at the caregiver’s household or at the health facility, depending on their preference and ease of access for the caregiver. When possible, research staff obtained the phone number of the caregiver at the time of consent to arrange interviews. If, after repeated phone and text messages, the study team was unable to contact the caregiver, a home visit was attempted at least once. Caregivers who could not be reached after three failed attempts at contact or who moved outside the study sites were classed as lost-to-follow-up. IDIs were carried out by trained local social science researchers, four women and two men, and conducted in the local language; Luo (Homa Bay and Muhoroni), Luhya (Funyula) and Swahili (for those who could not consent in the local language). Informed consent was obtained from the caregiver prior to each interview including consent for the interview to be recorded and for the use of anonymous quotes. Audio recordings of the IDIs were transcribed by the field researcher in the local language and subsequently translated into English. Transcripts were anonymised and labelled with a unique participant ID, indicating the study community and round of data collection.

### Data management and analysis

Transcripts from the caregivers in the cohort from all three rounds of data collection were imported into NVivo (QSR International) version 12 for coding and analysis. Initial coding of transcripts was done in line with the overall objectives of the QLS and consisted of 1) perceptions about malaria and vaccinations, 2) information sources and messaging related to the malaria vaccine, 3) malaria vaccine uptake experience – including information provided at visit and side effects, and 4) adherence to the 4 doses. In addition, three published frameworks were used to explore 1) access [19] (constructs include accessibility, affordability, accommodation, availability, and attitude), 2) acceptability [20] (constructs include affective attitude, burden, ethicality, intervention coherence, opportunity costs, perceived effectiveness, and self-efficacy) and 3) vaccine hesitancy (3C framework - confidence, complacency, convenience) [11]. Themes and sub-themes were added inductively as they emerged from the data. Data were coded by one researcher (JHo) and coding validation sessions with the data collection team (GO and TB) were done after each round of data collection to discuss findings, situate the data contextually and reach consensus on differing perspectives. Data saturation was discussed after each round of interviews to identify emerging themes and meanings that required further exploration in subsequent interviews. By the end of the third round of interviews the consensus was that data saturation had been reached as no new meanings had been identified.

**Fig. 1:**
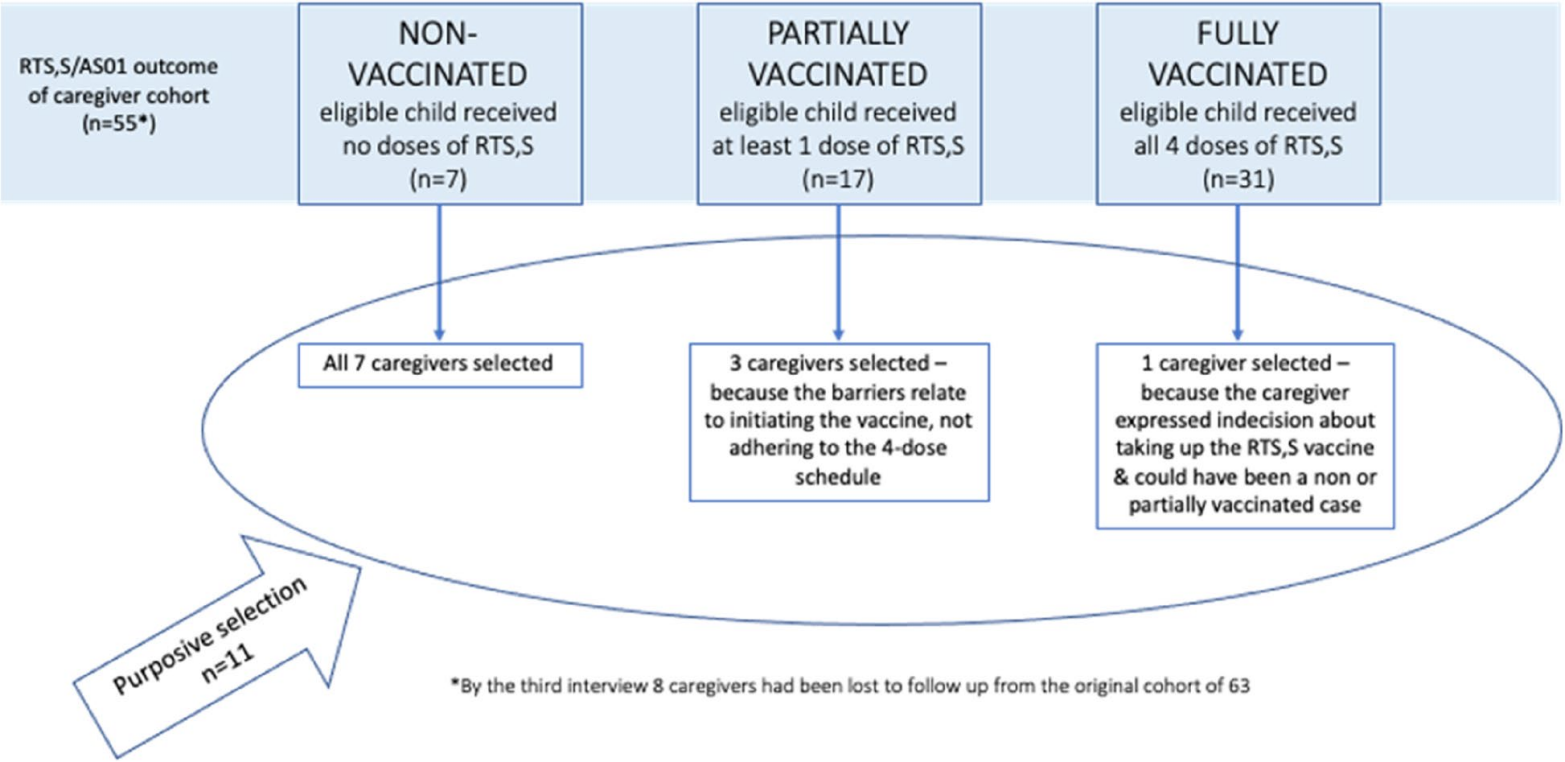
Caregivers of non, partially, and fully vaccinated children included in this sub-analysis

Of the 55 caregivers interviewed at all three time points, the data from a sub-set of 11 caregivers were purposively selected for further analysis to explore factors influencing *uptake* of the malaria vaccine at each time point, including identification of barriers and possible motivators. All seven caregivers of eligible children who were *non-vaccinated* (did not receive any dose at any time during the study period) were included. Of 17 caregivers of *partially vaccinated* children (received at least one dose during the study period but did not complete the full four doses) three were included because their experiences related to the initiation of the vaccine. In addition, one caregiver of a *fully vaccinated* (received all four-doses) child was purposively included because they were the only caregiver in the cohort to explicitly express ‘hesitant attitudes’ regarding uptake of the vaccine, especially to understand how such attitude did not result in a partially or unvaccinated child (Fig. 1).

Two different analyses of *uptake* were performed in this sub-set of caregivers: 1) a thematic exploration of the barriers and potential motivators to *uptake* across the caregivers, and 2) a trajectory analysis to understand how those factors may have changed over *time*. First, the transcripts at all three time points for these selected caregivers were re-coded. Themes and sub-themes were coded inductively and organised around the 5A taxonomy (Table 2) to 1) understand how they relate to the root causes of under-vaccination - *awareness, acceptance, access, affordability* and 2) identify potential instances of *activation*. Cross cutting themes among the uptake barriers and potential motivators were identified across the sub-set of caregivers. Secondly, a trajectory analysis was conducted using a time-ordered, sequential matrix for each caregiver to map the factors influencing uptake across time and identify the underlying factor(s) contributing to the partial or non-uptake of the vaccine at each time point [15]. Narratives were developed to highlight the unique and nuanced attributes of each caregiver’s experience with taking up the malaria vaccine [21]. Anonymised quotes were used to support the analysis and illustrate the underlying factors influencing the uptake experience of the caregivers.

**Table 2:** The 5A domains for root causes of under vaccination

**5A Domains**	**Potential factors related to each domain**
Awareness	Knowledge of vaccines & schedule
	Availability of information
	Consideration of vaccination
Affordability	Direct & indirect costs associated with vaccination (i.e., transportation to facility)
Acceptance	Perceived safety (including side effects) & efficacy of the vaccine
	Perceived risk, vulnerability to disease
	Individual characteristics: health beliefs, omission bias, trust, past behaviours
	Social context: social responsibility, peer influence, HP influence
Access	Geographical location
	Location of birth
	Contact with health services (regular vs. infrequent)
	Convenience of access
Activation	Factors that helped nudge a person to take up a vaccine

Thomson A, Robinson K, Vallée-Tourangeau G. The 5As: A practical taxonomy for the determinants of vaccine uptake. Vaccine. 2016;34(8):1018–24

The 5A taxonomy developed by Thomson et al. [22] was used to support this sub-analysis because it comprises broad domains that capture the root causes of under-vaccination. In addition, this taxonomy includes a novel fifth dimension *activation –* defined as *‘actions that nudge people who intend to get vaccinated to take up the vaccine’* [22]. This domain provided an opportunity to examine the experiences of caregivers in this sub-analysis whose children had received any doses of the malaria vaccine to understand if uptake of the vaccine may have involved an external nudge, which could offer valuable insights into how some barriers were overcome.

### Patient and public involvement statement

The views and experiences of caregivers were sought as participants in this study, they were not involved in the design of the study. However, they were invited to discuss and validate the study findings prior to the final analysis and dissemination of preliminary findings to key stakeholders.

## Results

The characteristics of the 11 caregivers and the number of malaria vaccine doses received by the eligible child prior to each interview are provided in Table 3. The main barriers to uptake for caregivers with partial or non-vaccinated children were captured by the 5A domains, and potential motivators were identified, which are used to frame the results (Table 4). In addition, seven narratives are presented throughout the results to illustrate how the factors influencing uptake 1) relate to the root causes of under-vaccination (5As), 2) are interrelated and change over time.

**Table 3:** Characteristics of the caregivers and RTS,S/AS01 doses received by their child per interview

ID	Community	Gender	Age	Education level	Total number of children	Pentavalent doses received by 3^rd^ interview (out of 3)	Measles doses received by 3^rd^ interview (out of 2)	RTS,S/AS01 doses received by child/interview			Narrative presented in manuscript
								1^st^ interview	2^nd^ Interview	3^rd^ Interview	
Non-vaccinated *n* = 7											
37	C11	F	33	Primary incomplete	9	2	0	0	0	0	Narrative 2
38	C11	F	27	Primary complete	4	2	1	0	0	0	Narrative 1
20	C12	F	26	Secondary incomplete	6	n/a	n/a	0	0	0	Not shown
43	C12	F	42	Primary complete	8	3	1	0	0	0	Narrative 3
23	C14	F	36	Secondary incomplete	6	3	2	0	0	0	Narrative 5
61	C18	F	31	More than secondary	2	3	2	0	0	0	Not shown
63	C18	F	30	Primary complete	7	3	1	0	0	0	Not shown
Partially vaccinated *n* = 3											
15	C16	M	26	Primary complete	4	3	1	1	1	1	Narrative 6
42	C12	F	36	Primary incomplete	7	1	0	1	2	2	Not shown
56	C17	F	26	Primary incomplete	4	3	1	0	2	2	Narrative 4
Fully vaccinated *n* = 1											
32	C16	F	26	More than secondary	1	3	2	1	3	4	Narrative 7

**Table 4:** Caregiver themes across the 5A taxonomy and how they changed over time

**5A domains**	**Key themes from caregivers**	**Changes over time**
**Awareness**	• Low awareness of the malaria vaccine • Incorrect information about eligibility or delivery point of the vaccine	➔ low awareness led to initial delays to get dose-1 but awareness of the vaccine grew over time ➔ incorrect information about eligibility and the delivery point persisted over time and led to delays that pushed children beyond eligibility
**Acceptance**	• Hindered by fears related to side effects from previous immunisations - infants get ‘too many’ injections • Hindered by concerns that the malaria vaccine is new and being tested • Facilitated by attitudes that vaccines in general are good and effective • Facilitated by perception that the new malaria vaccine is effective	➔ experiences with previous side effects initially delayed dose-1 uptake but this fear subsided over time due to perceived lived benefits of the vaccine & positive messages from peers about the vaccine ➔ malaria vaccine specific concerns caused initial delay, but fears were overcome due to repeated messages at the health facility ➔ attitude that vaccines are generally good persisted across all interviews ➔the perception that the malaria vaccine is effective and protects children grew over time
**Access**		
*** Individual level (I)***	• Burden of getting to the health facility was an access barrier • Competing life events delayed uptake of the vaccine	➔ inconvenience of getting to the facility (with multiple infants or while pregnant) delayed uptake of dose-1 ➔ work, travel away or illness contributed to initial delays in getting dose-1
*** Health system (HS)***	• Negative provider attitudes discouraged attendance at immunisation services • Immunisation services not available (provider strikes, service schedule) • Barriers related to vaccine stockout at the facility-level	➔ delays persisted for some caregivers because of fears that their providers would scold them for being late, missing doses or not having their MCH^a^ booklet ➔ unavailable services or vaccines persistently frustrated caregiver attempts to take up the vaccine
**Affordability**	• Transportation costs to get to the facility was an uptake barrier	➔ having to find or not having transportation money increased the burden of getting to the health facility
**Activation**	• Promoted messages about the new malaria vaccine at health facility • Screening by provider that captures missed immunisations provided new opportunities to initiate dose-1	➔ messages about the malaria vaccine encouraged uptake of dose-1 ➔ some eligible children received dose-1 because of provider screening for missing immunisations during a facility visit for other reasons

^a^*MCH booklet* Mother and child health booklet

### Awareness

**Fig. 2:**
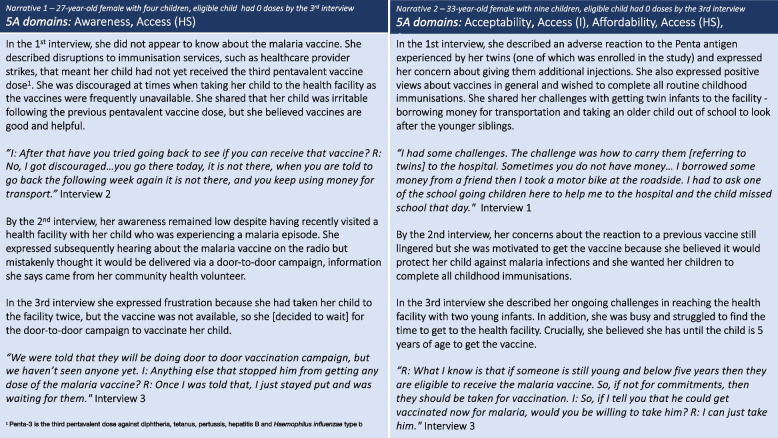
Caregiver narratives 1 and 2

Initially, lack of awareness and incorrect information about the malaria vaccine delayed several caregivers from taking their eligible children to receive dose-1. Some caregivers reported that, prior to the interview, they had either not heard about the vaccine or had heard of it but were not confident they had understood the information. In some cases, initial incorrect information about the malaria vaccine – either where it was available or eligibility – contributed to uptake delays that meant the child missed the eligibility window for dose-1. In *narrative 1*, the caregiver demonstrated low awareness about the vaccine from the first interview and persistently reported incorrect information about how the malaria vaccine would be delivered (she reported being told the vaccine would be delivered by door-to-door campaign), which in conjunction with health system access barriers led to complacency whereby she decided to wait for the (non-existent) campaign. In *narrative 2*, the caregiver was apprehensive about additional vaccines due to a previous reaction to the Pentavalent antigen and the inconvenience of traveling to the facility with two young infants, and yet she was motivated to vaccinate her child. However, incorrect information about the eligibility age contributed to her complacency, as she believed her child could initiate the malaria vaccine any time up to the age of 5 years (Fig. 2).

### Acceptance

**Fig. 3:**
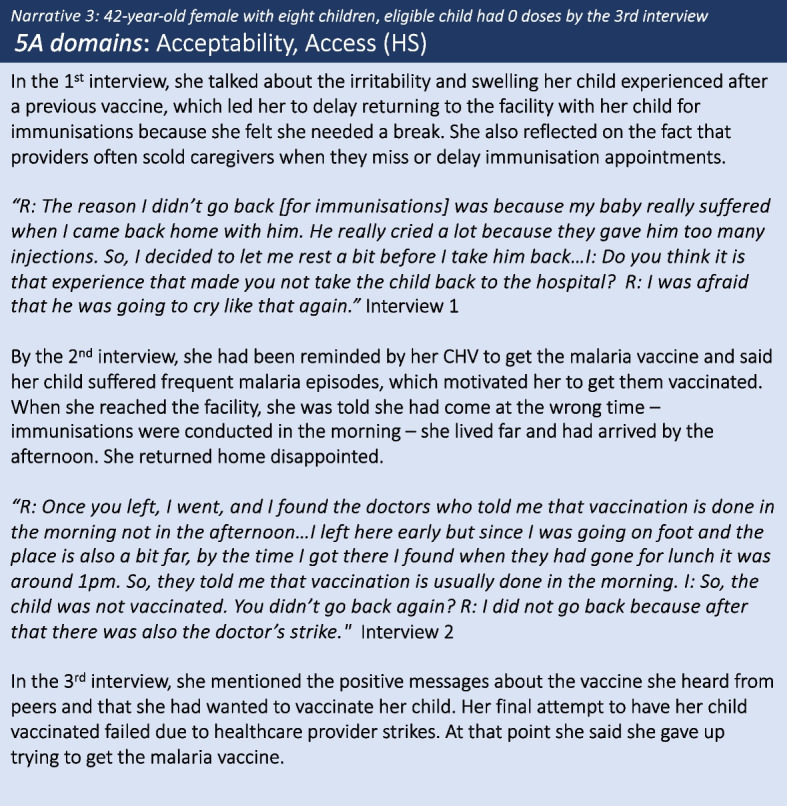
Caregiver narrative 3

Negative experiences with vaccine side-effects following previous immunisations (Pentavalent, Bacillus Calmette-Guerin (BCG^1^)), such as having an irritable child or swelling and abscesses at the injection site, were cited as reasons for delaying further vaccinations, including the malaria vaccine. Caregivers described the challenges associated with having an irritable, unwell child for several days post vaccination. Some caregivers linked the side-effects to the child having been given ‘too many’ injections. Despite the unpleasant side-effects, these caregivers also believed vaccines helped protect their children against harmful diseases and did not feel side-effects would ultimately deter them from seeking further vaccinations. Only one caregiver expressed concerns specifically related to the malaria vaccine being new and still in the testing stages (see narrative 7). In both narrative 2 and 3, the caregivers attributed some initial delay to a previous negative experience involving a routine childhood vaccine. However, in subsequent interviews they expressed motivation to vaccinate their child because of the perceived protection offered by the vaccine, specifically in reducing malaria episodes and severity of infection. Having overcome their initial hesitation related to *acceptance,* uptake was ultimately de-railed due to individual-level or health systems *access* barriers. In *narrative 3*, initial motivation to get the malaria vaccine after hearing positive messages from peers turned to disappointment after two failed attempts to have her child vaccinated at the health facility. In the final interview the caregiver reported she had eventually given up (Fig. 3).

### Access and affordability

**Fig. 4:**
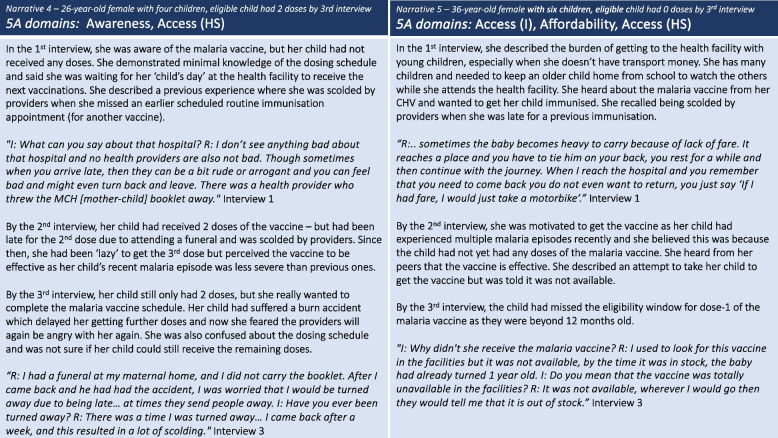
Caregiver narratives 4 and 5

Two distinct levels of *access* barriers were identified at the individual- and health system-level. Individual-level *access* barriers, including the burden of getting to the facility whilst pregnant or with multiple infants and having to keep an older child home from school to watch over younger siblings, delayed the uptake of the vaccine for some caregivers. These barriers were at times exacerbated by *affordability* – such that had they had the transport fare, that burden may have been reduced. Additionally, competing life events, such as travel away to attend funerals or family illness led to missed appointments and contributed to uptake delays. *Narrative 4* reveals how these delays were often compounded by fears of being scolded by healthcare providers (health system access barrier) for delayed or missed appointments, a fear that persisted across all three interviews and served as a strong deterrent against seeking immunisation services. For nearly all caregivers in this study, health system barriers were present. *Narrative 5* illustrates the dynamic between individual level and health system *access* barriers, exacerbated by the indirect costs of getting to the facility, which in some cases pushed children beyond the eligible age. Caregivers expressed their frustration at having reached health facilities only to find that vaccines were not available, immunisation services were closed, or the providers were on strike (Fig. 4).

### Activation

**Fig. 5:**
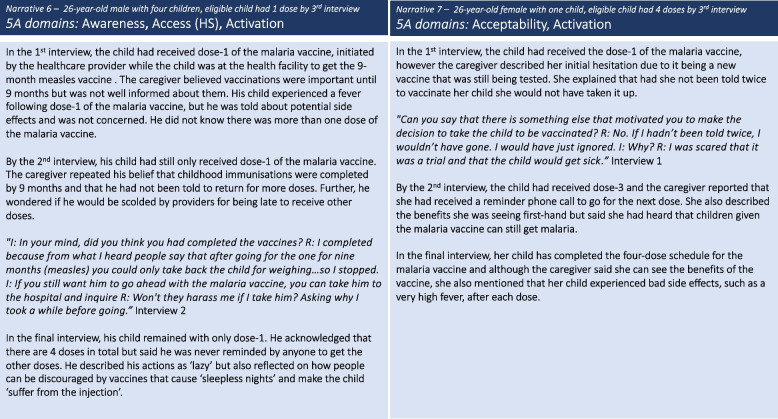
Caregiver narratives 6 and 7

*Activation* factors, such as attending the health facility for another reason, created opportunities for eligible children to receive dose-1 without the caregiver having deliberately sought it. Activation factors were identified among caregivers whose child had received at least one dose of the malaria vaccine. These factors included providers screening for eligible children when they attended the health facility for other reasons such as routine growth monitoring, the first dose of the measles vaccination at 9 months, or treatment seeking. However, despite this opportunity to initiate the dose-1 of the malaria vaccine, it did not always result in adherence to the full four doses. This was due to low caregiver awareness coupled with inadequate information by providers regarding the number of doses or return dates for subsequent doses, leading some caregivers to apparent complacency, as illustrated in *narrative 6*. Over the three interviews, few caregivers expressed concerns relating to the malaria vaccine specifically and as noted previously, confidence in the benefits of the vaccine grew over time, related to the reduced frequency and severity of malaria infections. However, as illustrated in *narrative 7*, initial apprehension about the malaria vaccine being new and still undergoing testing led one caregiver to hesitate. This caregiver’s experiences suggests that activation in the form of repeat messaging whilst at the health facility can motivate some to take up the vaccine, even when concerns have been expressed (Fig. 5).

## Discussion

This study identified barriers and potential motivators that influenced uptake of the RTS,S/AS01 malaria vaccine among caregivers in the context of the pilot programme in western Kenya. The longitudinal cohort approach enabled a nuanced understanding of the dynamic influences that shaped caregiver experiences and, for some, hindered uptake of the first dose. Crucially, the longitudinal analysis exposed a complex interplay between initial delays by the caregiver exacerbated by health system barriers. Reasons for initially delaying uptake of the vaccine included lack of awareness about eligibility and where to access the malaria vaccine, the burden of reaching the health facility due to individual caregiver factors, and apprehension about side-effects due to negative experiences from previous routine childhood vaccines. However, as confidence in the effectiveness of the malaria vaccine grew due to word-of-mouth and the perceived benefits in children who had received the vaccine, caregivers were motivated to get their child vaccinated. Importantly, some caregivers overcame personal barriers only to be discouraged by persistent health system constraints, such as unavailable vaccines, poor healthcare provider attitudes and provider strikes, resulting in the non-uptake of the first dose despite repeated attempts.

Initial delays by caregivers played a major role in the partial or non-vaccination of some children due to the limited eligibility window to initiate the first dose (6- ≤ 12 months) of the malaria vaccine. Lack of caregiver awareness in the first interviews was likely due to the recent launch and inadequate social mobilisation for the malaria vaccine pilot (Jenny Hill, personal communication). Fear of side effects to previous antigens (Pentavalent or BCG) prompted some caregivers to delay uptake of the first dose of the malaria vaccine. Safety concerns or lack of confidence in immunisation programmes generally, leading to delay or refusal of vaccinations, have been reported elsewhere [23–25]. Uniquely, this study identified ‘having an irritable child’ as a common theme leading some caregivers to want to ‘take a break’ from immunisations. Post-vaccination irritability in children was said to negatively affect work and sleep, in addition to which caregivers did not want their child to suffer. Contrary to earlier studies exploring community perceptions to the anticipated malaria vaccine in Kenya and Tanzania [7, 8], safety concerns related to the vaccine were uncommon and caregivers were generally appreciative of the protection afforded by vaccinations more broadly. The burden of getting to the health facility for vaccinations, such as needing to take older children out of school to care for younger siblings, also delayed uptake and may partially explain the association between lower immunisation uptake and family size reported in other studies [26, 27]. Additionally, competing life events such as pregnancy, work, illness, and travel contributed to delays and are well documented barriers to immunisation uptake generally [6]. Significantly, most caregivers eventually overcame these initial personal barriers and attempted to vaccinate their child, motivated primarily by the positive perception of the malaria vaccine coupled with the profound burden of malaria on their households.

Persistent health service access barriers were a major contributor to some children missing the eligibility window for dose-1 of the malaria vaccine. Caregivers overcame barriers to reaching the health facility only to find immunisation services or vaccines were not available, a widely reported immunisation service constraint [6]. Though service disruptions due to COVID-19 were reported at the study sites, this was not identified by caregivers as a barrier. Providers chastising caregivers for being late for immunisation appointments, or having missed a dose, emerged as a significant deterrent to attending immunisation services. Negative provider attitudes was anticipated as a potential constraint to uptake of the malaria vaccine prior to pilot implementation in Kenya [7] and to immunisations in general [28, 29] and underscores the need to improve interpersonal communication skills among providers. Further, the quality of immunisation services has been found to be a key component of sustained vaccination coverage more broadly [30]. Insights from this study suggest that the children of infrequent health service users are at particular risk of not getting the malaria vaccine. First, because they have reduced opportunities to receive key messages about new vaccines, but also because they have fewer chances to be screened for eligibility. Health system utilisation is a significant determinant of full immunisation [31]. This study provides some insight into how contact with the health facility could be an important activation factor for the uptake of dose-1 of the malaria vaccine, through repeated messaging and opportunities for providers to screen for eligible children. The narratives of several caregivers in this study exposed a worrying cycle whereby those who experienced repeated discouraging encounters with the health system, indicated diminished confidence in the immunisation services, which reinforced infrequent use. Repeated failed attempts to get dose-1 of the malaria vaccine was a key driver of caregiver complacency and led some to give up on it altogether, contributing to low coverage of children with all four doses completed (Jenny Hill, personal communication).

The experiences of caregivers, and the behaviours and barriers that lead to some children being partially or non-vaccinated, can be misinterpreted. Bedford et al. noted that vaccine hesitancy is often used inaccurately to explain under- or partial-vaccination when causes are in fact related to ‘pragmatics, competing priorities, access or failures in service’ [14]. Consistent with a previous review on potential implementation hurdles [9], caregivers in this study were broadly accepting of the new malaria vaccine. Only one caregiver expressed ‘hesitant attitudes’ with regards to the vaccine owing to the fact it was still being tested (referring to the pilot evaluation), but all caregivers faced challenges in taking up the vaccine, many of which could be miscategorised as *complacency* or *convenience*. However, when understood in the context of the caregiver’s experiences over time, it is a combination of individual and health system access barriers that underpinned their behaviour. To effectively design interventions that optimise uptake there must be a clear distinction between the barriers that affect access and the factors that drive hesitancy [13]. At various time points caregivers in this study may have appeared complacent, however what emerges is that repeated encounters with health system constraints fuelled that complacency, and over time contributed to non-uptake of the vaccine. As such, the appropriate intervention is adequately resourced, flexible, and quality immunisation services to improve confidence, together with continuous information and messaging on the benefits of the malaria vaccine to promote adherence to all four doses.

The failure to understand the reasons behind caregiver behaviour could lead to unsuccessful interventions. For example, understanding caregivers’ concerns regarding side effects are important. Findings from this study suggest that effective provider communication about potential side effects and how to manage them may reduce the burden on both caregiver and child, increase confidence in the vaccine and reduce future delays in vaccine uptake. Some health providers reported prescribing paracetamol alongside immunisations to help reduce potential side-effects (personal communication, George Okello); further research may be warranted on whether the provision of paracetamol post-vaccination leads to increased caregiver confidence in vaccines and improves uptake and adherence. Similarly, delineating between the burden some caregivers face in reaching the health facility due to large family sizes or competing life events and health service access barriers are crucial in prioritising effective interventions to boost coverage. Strategies that facilitate ease of access to immunisation services and the malaria vaccine, such as outreach services and community catch-up campaigns, would eliminate some of the burden on caregivers and could mitigate the impact of other health system barriers.

### Strengths and limitations

The longitudinal design enabled a temporal exploration of the factors influencing uptake. However, there are some important limitations of this approach. Firstly, the small sample included in this sub-analysis means that other key factors and contexts affecting uptake could have been missed. Secondly, this study involved repeated contact between the caregivers in the cohort and the research team over the course of a 2-year period, thus these caregivers received additional prompting to take up the vaccine compared with caregivers outside the study setting. We detected evidence of caregiver’s behaviour linked to the reminders and encouragement prompted by the repeated visits of the field staff. To counter this the research team engaged in reflexive exercises to understand how their presence might have influenced the caregiver experience and how to interpret findings in this context. For instance, it was difficult to objectively assess the level of caregiver awareness about the malaria vaccine after the first interview because of the additional information provided through interaction with the research team. To examine researcher influence more fully, a cross sectional survey of caregivers was included in the final round of data collection for comparison with findings from the cohort (results to be published elsewhere).

## Conclusions

The experiences of caregivers with partially or non-vaccinated children in accessing the newly launched malaria vaccine in western Kenya demonstrate that the factors influencing uptake were not static but dynamic over time. Initial lack of awareness and misinformation about eligibility and availability of the new vaccine, negative experiences following previous immunisations, and the burden of getting to health facilities resulted in delayed uptake for some caregivers. Ultimately, health system constraints, including poor healthcare provider attitudes, lack of services due to strike action and stock-outs, meant that some children missed the eligibility window for the first dose. Community-based delivery strategies offering additional opportunities for caregivers to access the vaccine would mitigate some health system constraints, together with interventions to improve inter-personal communication skills among healthcare providers.

### Supplementary Information

**Additional file 1: Table S1.** The Standards for Reporting Qualitative Research (SRQR) checklist.

## Data Availability

The datasets generated and/or analyzed during the current study are not publicly available to maintain the anonymity of the participants. The data can be made available from the corresponding author on reasonable request.

## Abbreviations

SSA: Sub-Saharan Africa
WHO: World Health Organisation
QLS: Qualitative longitudinal study
MVIP: Malaria vaccine implementation programme
CHV: Community health volunteer
IDI: In-depth interview

## Footnotes

1 BCG is the Bacillus Calmette-Guérin vaccine against tuberculosis.
