# Supplementary material for: RTS,S/AS01 malaria vaccine pilot implementation in western Kenya: a qualitative longitudinal study to understand immunisation barriers and optimise uptake
Source: BMC Public Health. 2023 Nov 18;23:2283. doi: 10.1186/s12889-023-17194-2 (PMC10657022; doi:10.1186/s12889-023-17194-2)
Supplement: Supplementary file 1 — Additional file 1: Table S1. The Standards for Reporting Qualitative Research (SRQR) checklist. [file 12889_2023_17194_MOESM1_ESM.pdf]

Table S1: The Standards for Reporting Qualitative Research (SRQR) checklist

Title and abstract		Page number
1. Title	Concise description of the topic of study	P1
2. Abstract	Includes background (& aim of study), methods, results, conclusions	P2
Introduction		
3. Problem formulation	Description of phenomenon being studied, review of empirical work	P4
4. Purpose or research question	Purpose of the study/specific objectives	P4
Methods		
5. Qualitative approach and research paradigm	Qualitative longitudinal study and rationale for study design	P5
6. Researcher characteristics and reflexivity	Description of researchers, data collectors	P1, P6-7
7. Context	Description of study sites and contextual factors	P5-6
8. Sampling strategy	Selection of study sites; caregiver selection for in-depth interviews	P5-6
9. Ethical issues pertaining to human subjects	Ethics approval from four ethics committees; informed consent from participants	P14
10. Data collection methods	Description of data collection (in-depth interviews)	P6-7
11. Data collection instruments and technologies	Details of discussion topic guides	P6
12. Units of study	Number, type and characteristics of participants	P6, P8, P17
13. Data processing	Management and processing of data	P7
14. Data analysis	Description of coding and analysis – thematic and trajectory; framework used to support analysis	P7-8
15. Techniques to enhance trustworthiness	Description of coding validation discussions and consensus building among the research team	P7
Results/Findings		
16. Synthesis and interpretation	Main findings reported	P8-11
17. Links to empirical data	Caregiver narratives	Figures 2-5
Discussion		
18. Integration with prior work, implications, transferability, and contributions to the field	Summary of main findings, situating findings along existing literature; discussion of how these findings contribute to the field of inquiry	P11-13
19. Limitations	Limitations of the study, generalisability of the findings	P13
Other		
20. Conflicts of interest	None to declare	P14
21. Funding	WHO, GAVI, the Vaccine Alliance, The Global Fund, Unitaid	P14
